# A Narrative Review of Adverse Event Detection, Monitoring, and Prevention in Indian Hospitals

**DOI:** 10.7759/cureus.29162

**Published:** 2022-09-14

**Authors:** Snehil Verman, Ashish Anjankar

**Affiliations:** 1 Department of Community Medicine, Jawaharlal Nehru Medical College, Datta Meghe Institute of Medical Sciences, Wardha, IND; 2 Department of Biochemistry, Jawaharlal Nehru Medical College, Datta Meghe Institute of Medical Sciences, Wardha, IND

**Keywords:** artificial intelligence techniques, computerized surveillance, medication errors, adverse drug reactions, adverse events, electronic health records, detection, prevention, outcomes

## Abstract

An adverse event is any abnormal clinical finding associated with the use of a therapy. Adverse events are classified by reporting an event's seriousness, expectedness, and relatedness. Monitoring patient safety is of utmost importance as more and more data becomes available. In reality, very low numbers of adverse events are reported via the official path. Chart review, voluntary reporting, computerized surveillance, and direct observation can detect adverse drug events. Medication errors are commonly seen in hospitals and need provider and system-based interventions to prevent them. The need of the hour in India is to develop and implement medication safety best practices to avoid adverse events. The utility of artificial intelligence techniques in adverse event detection remains unexplored, and their accuracy and precision need to be studied in a controlled setting. There is a need to develop predictive models to assess the likelihood of adverse reactions while testing novel pharmaceutical drugs.

## Introduction and background

Adverse drug events (ADEs) or adverse drug reactions (ADRs) are hazardous to patients and contribute to an increase in morbidity and overall mortality [1]. ADRs are difficult to identify as they are seen in certain groups of people in some conditions and may be seen after long-term exposure [2]. This is why all drugs, before their release into the market, undergo rigorous clinical trials to discover potential ADRs before selling the products but are limited in numbers. As a result, post-marketing surveillance is required to help discover adverse drug reactions after the drugs are available on the market [3]. The per-hospital admission prevalence for adverse events varies from 2.9% to 16.6% [4-6]. There is a need to identify interventions for detecting and reducing medical errors and adverse events. The traditional ADR classification includes type A (pharmacologic) or type B (idiosyncratic) reactions [7]. The former makes up to 90% of all ADRs and is known due to the drug's pharmacological properties [8,9]. The latter is related to hypersensitivity reactions and can account for 10-15% [10]. As per expert opinion, most of this could be related to inflammation or immunologic mechanism. The onset of action needs to be studied (immediate vs delayed).

Immunologic drug reactions are classified as Type I, II, III and IV. Type I reactions usually start within an hour and are related to IgE antibodies [11]. But some reactions can appear after an hour if the drug is given orally, and food also affects the absorption of the drug. Among delayed reactions are those that arise after an hour, most of which begin after six hours and some after many days of treatment. Here, antibodies (IgG or IgM) are directed against cellular antigens, resulting in cellular destruction and tissue damage [7]. Type I reaction signs can be due to vasoactive mediators released by mast cells [3]. The most typical reaction is a rash, and the most severe is anaphylaxis. The timing of onset of Type 1 reaction is rapid but varies with the presentation. Type II and Type III are rare and are mediated by IgG/IgM. Type III reactions are mediated by antigen-antibody complexes and can present as drug fever. Type IV reactions are not mediated by antibody response and are delayed; hence are termed delayed-type hypersensitivity reactions [12].

When an event is confirmed, it can be dealt with in one of three ways: by administering unrelated medication, administering a related medication, and desensitizing (stopping) the culprit drug [13]. Risk factors for drug allergy include genetic factors, repeated drug exposure, and the presence of certain diseases like acquired immunodeficiency syndrome. The rates of hospitalization and the number of patients dying due to ADRs are gradually increasing. Hence, it has become challenging for healthcare providers to resolve this on priority. The main goal of ADR monitoring is to identify the ADR that has occurred, detect the associated risks, and reduce future harm to patients. Thus, this is a lifelong process for any drug. A computer-assisted publication search of MEDLINE was performed on May 9, 2022, to identify articles, guidelines and recommendations in the English language from 1980 to 2022 for adverse events, which were later critiqued and a narrative review was written. After mentioning the types of ADR and their various impacts, this narrative review focuses on the currently available methods for detecting adverse events, and reviews their advantages and discusses their limitations and the interventions to treat these events.

## Review

Methods for detection of adverse events

The methods are broadly classified into hypothesis-generating and hypothesis testing [14]. Voluntary reporting (physician self-reporting) forms a small portion of ADR information. [15]. This identifies the lowest number of errors compared to others. Medical record or chart review is a more systematic method than voluntary reporting but is labour-intensive and time-consuming [16,17]. Automated surveillance detects many events not captured by other systems [18]. Direct observation is an effective method but the most expensive. Computerized detection helps to identify a possible adverse event that can be tackled by timely human intervention. This can be effective by only screening the highly focused charts and excluding a large set of unnecessary data. Reports by patients and family members are considered of modest benefit and are reserved for research studies and clinical trials [19,20]. Table 1 mentions the detection methods and their advantages and limitations.

**Table 1:** Methods for detecting adverse drug reaction

Source	Advantages	Limitations
Articles in peer-reviewed journals	Simple, easy to obtain	Depending on individuals’ perceptions and judgement, may detect only everyday events.
Voluntary reporting (pharmacists, prescribing doctors, drug manufacturing companies)	Effortless and easily available	Reporting bias is often seen Details may be elementary, unrecorded, or underreporting.
Retrospective studies	Easy to perform and data already available, no need for subject’s participation	Suitable only for association and not causation; follow-up events, if any, may be missed.
Intensive event monitoring	Easily organized	Selected subjects studied for a limited time.
Case-control studies	Good for assessment	New effects are not studied; they are not economical.
Population statistics	A large group of people can be studied at a time.	Coordination becomes difficult; information may be of poor quality.
Meta-analysis	Utilizes data from a study that has already been performed	Reported studies may have a high degree of heterogeneity, unpublished data not included or need to be separately obtained for assessment.

Causality assessment in ADRs

**Figure 1:**
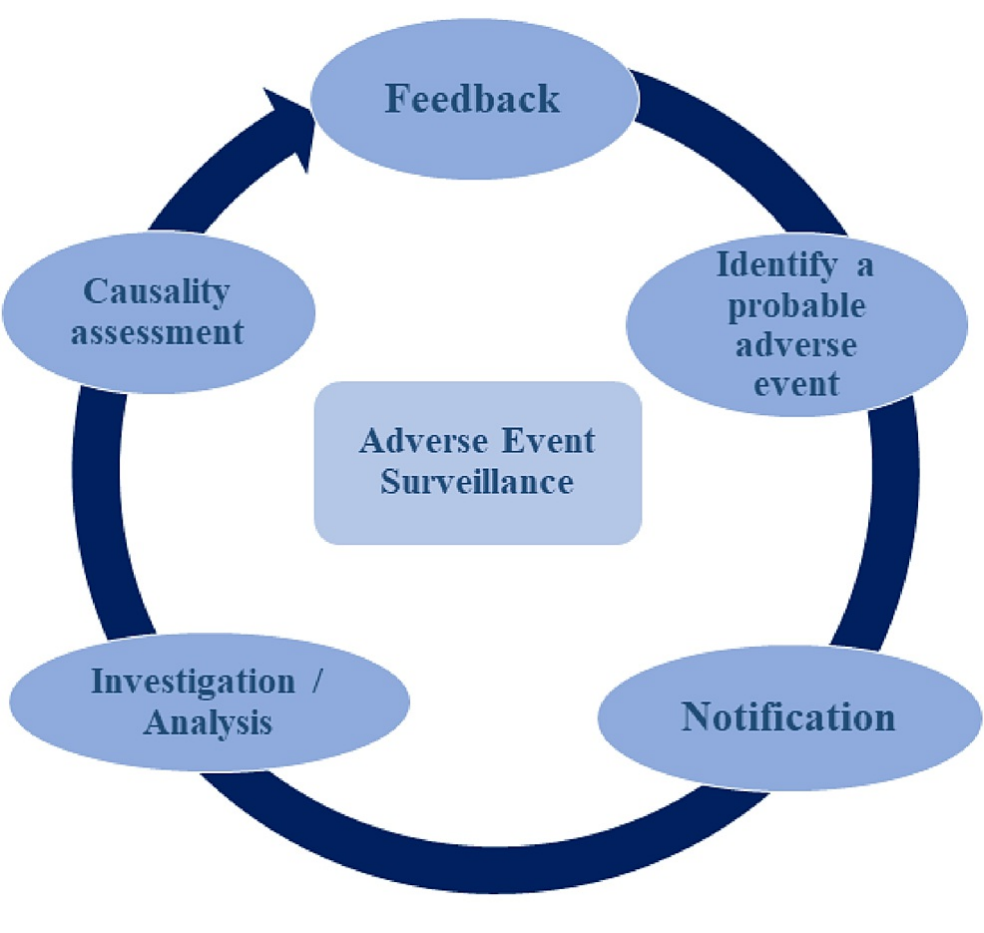
Mechanism of adverse event surveillance

It is vital to determine the causality and relationship of an event to drug use. The Naranjo ADR Probability Scale was developed in 1981 [21]. The probability of an ADR is based on its characteristics, the rater, the quality of the information, and the scale used. Initially, the World Health Organization-Uppsala Monitoring Centre (WHO-UMC) scale was used to study ADR, which has now been replaced by the Naranjo ADR Probability Scale, which appears to be more reliable and predictable due to its wide range of questions [22]. Medical records need to be reviewed and clinical history should be obtained whenever feasible (Figure 1). Allergen skin testing for immediate reactions and markers of anaphylaxis should be done. 

Managing ADRs and the need for interventions

**Figure 2:**
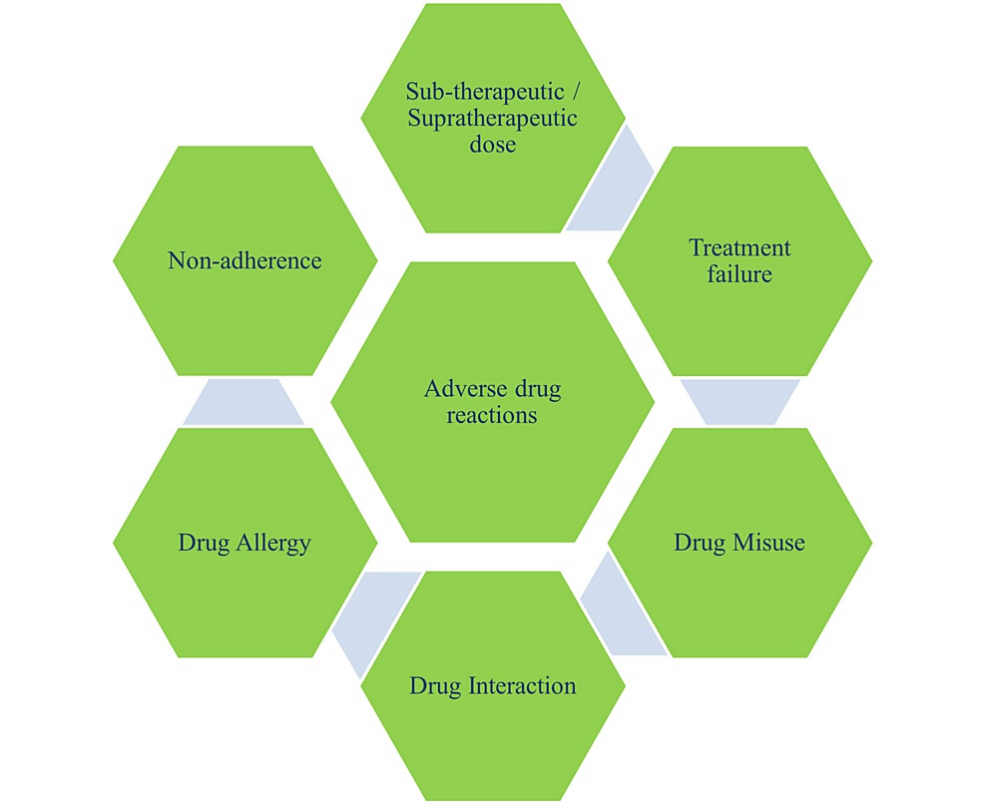
Contributors to adverse drug events

Adverse events start with the reporting of such events by a patient to the treating doctor or to the pharmacist [23]. Adverse event reporting starts with suspicion by the treating doctor that an unexpected adverse event may be causally related to the drug being used (Figure 2). Such an adverse event should be reported to the Food and Drug Administration and the drug manufacturer when feasible [24]. Medication errors need to be reduced to manage overall ADRs. [25]. This can be taken care of during prescribing, administration, and monitoring. Provider and system-based interventions are used to prevent ADR The provider-based approach is more appropriate by looking at the medication list at each patient encounter. Safe prescribing guidelines need to be followed for prescribing high-risk drugs. Patients or specific populations, such as geriatric, pediatric, pregnant women, and patients with substance abuse, may be over-susceptible to ADRs. Additional care should be taken while prescribing drugs to these patients [26]. The unnecessary use of drugs should be stopped (deprescribing). This is also as essential as other approaches to overcome ADR. Any new symptom/complaint must be associated with the concomitant use of a new drug. These are preventable ADRs, and one should avoid treating the side effects of one drug with the new medication. 

When prescribing a drug, the doctor should look for possible drug-drug interactions. The dosing needs to be adjusted based on age and creatinine clearance levels. Patients should be warned about the effects of likely non-adherence and how it should be dealt with. Computerized physician order entry has clinical decision support systems that help avoid unnecessary ADR when a patient is on multiple drugs, thereby preventing medication error. Educational programs may have a limited role in reducing medication errors. A list of allergies must be stored in a repository to prevent drug allergies. These systems will duly work if we understand the nature of mistakes and try to improve them [27].

The burden of ADR contributes to an overall increase in the cost of hospitalization, with a prevalence of 6.5%, with ADR contributing to 80% of hospitalization [28]. Studies on using artificial intelligence (AI) to leverage the effect of ADR are currently in their infancy and need more robust studies [29]. ADR. can cause disruptions in continuing therapy [30]. An adverse event is difficult to determine in routine clinical trials as the trial may be underpowered to detect rare events [31]. The trial may be short, the limited population may be exposed, and the trial may lack patient diversity. Some ADRs can be seen after several years after the drug has been on the market [32]. Developing countries like India need to be equipped with resources for monitoring drug safety and quality by setting up a sound pharmacovigilance system. Several challenges still need to be overcome before the explicit implementation of resources for adverse event detection and reporting. The doctors must be reassured that there will not be any legal proceedings against them for reporting any adverse event [33]. Not only this, but the doctors should also receive routine education and training to increase awareness through the dissemination of manuals and newsletters containing details of recent ADRs. The online ADR reporting form in India was initially available only in English, and regional languages have been recently added by the Indian Medical Association for ease of participation of local doctors. Unlike ADR, no device-related adverse event reporting database exists in India. The Central Drugs Standard Control Organisation (CDSCO) keeps finding new monitoring centres and has started to train its staff in hospitals and research centres in India [34]. These are audited to ensure the quality of reported cases should be uniform. Patient safety can be improved by early detection, prevention, and mitigation of the occurrence of an adverse event. The events identified by doctors are likely to be preventable. Several high-risk drugs are consistently associated with ADRs. The use of such medications should be reduced or needs to be replaced with other alternative medicines with better safety profiles. Unnecessary drugs should be discontinued [35]. The dose of drugs should be adjusted according to age and creatinine clearance levels. 

Underreporting of ADR has prompted the inclusion of all data sources for pharmacovigilance, including administrative claims and electronic health records, all the relevant medical literature, social media posts, and internet search logs that analyze multiple sources of details for ADR detection [36]. The incidence of ADE varies depending on the definition of events, clinical settings, and detection methods (e.g., chart review, voluntary reporting, direct observation, computerized surveillance) [37]. The earliest reports came from observational studies done in the United States in tertiary care institutes [38]. ADE is classified as before hospitalization, at hospitalization, and after discharge from the hospital [39]. Adverse events can occur in any hospital, including the emergency room, trauma wards, and operating rooms. Most commonly, it is seen in intensive care units and during weekends [40]. Whether an adverse event is reported depends on doctor attitudes, simplicity of completing the report, concern about liability, fear of punishment, and culture of the organization [41]. 

Medication reconciliation helps to prevent medication discrepancies by reviewing a patient's drug history and clarifying that the drugs and their doses are appropriate [42]. The medication ordered and those on the list must be cross-checked, documented, and communicated to the patient at discharge [43]. Bar codes mentioned on the drugs and wristbands help match patients and their medications during medication administration [44]. The privacy of hospital staff who report medication errors and follow-ups should be maintained to ensure the implementation of improvements. Older patients are highly vulnerable to ADR due to diminished physiologic reserve, multiple comorbidities, and the use of various drugs [45]. Studies have shown that older patients are four times more likely to be hospitalized for ADR than younger adults [46]. Once a drug allergy is identified, the patient should be educated regarding avoiding the drug, along with a written list of generic names of the culprit drug. This ensures that patients take adequate care and responsibility for their disease management.

In pregnant patients, physiological changes indirectly influence drug absorption, metabolism, distribution, and elimination [47]. Physiological changes during pregnancy can affect gastrointestinal function, thereby affecting drug absorption. Some drugs may have an increased half-life, thereby contributing to toxicity. The drug concentration in milk is proportional to that in maternal plasma. Certain drugs are excreted into the milk but rarely attain therapeutic levels [48]. However, infants need to be watched, and their mothers need to be educated. If the patient has pregnancy-related hypoalbuminemia, there would be decreased protein binding, and more free drugs would be available for excretion [49]. The dosage of medicines needs to be altered during pregnancy so that the therapeutic window is reached for maximum effectiveness. The drug should also not cross the maximum safe levels, leading to ADR development. 

Patient education can also help prevent certain adverse events. For example, certain drugs like immunomodulators need frequent blood tests to avoid drug toxicity. The optimal dose of immunosuppressants needs to be tweaked so that the drug remains at therapeutic levels and does not lead to toxicity [50]. This is usually seen in patients who undergo organ transplants, especially liver and kidney transplants. These patients need to do serial blood counts, and doctors need to advise these patients to follow up regularly. People start taking more medicines as they keep ageing. They should be warned to take drugs only if necessary and avoid unnecessary over-the-counter medications. There are associated psychological issues that some patients need to deal with. In such cases, it is challenging to manage patients on multiple drugs [51]. Doctors have the prerequisite expertise and knowledge to identify and treat ADRs. It is easy to miss specific ADRs when too many drugs are involved in a patient's therapy. There is no organized pharmacovigilance system in place at various hospitals in India compared to countries where electronic medical records make life easier for detecting ADR.

In the majority of the hospitals in India, the clinical pharmacy practice department takes control of the pharmacovigilance activities. The clinical pharmacist or a pharmacologist is always available to answer queries related to ADR in significant hospitals. Nurses are also involved in filling up A.D.R. forms at certain hospitals. Concomitant administration of different drugs can also lead to the development of ADR. However; it is difficult to understand the drug contributing to ADR when a patient is on polypharmacy. Patients' outcomes are affected when there is a frequent occurrence of ADR. Patients often start doubting the medication and may be wary of it as it may cause another ADR [52]. This affects adherence to therapy, and non-compliance leads to poorer patient outcomes. Hence there is an unmet need to identify appropriate ADR at every level of drug exposure in patients. Patients must be educated that unnecessary medications do more harm than good and will not improve overall health. Antibiotics and painkillers are the most sold drugs across the world [53]. Patients should be educated that these medications taken without medical supervision can cause adverse events.

AI can shorten the entire process of ADR reporting by reducing documentation workload [54]. The highly technical computerized systems might help fast-track ADR. monitoring and prevent adverse events. There is undoubtedly a bright future in this less traversed road that needs to be explored in future prospective trials. 

## Conclusions

Adverse events can be minimized by active monitoring, but it is challenging to eliminate them from practice. Optimizing patient safety requires the reduction of potential harm before and after the marketing of a drug. Identifying the exact benefit-to-risk ratio in a drug is necessary, and those drugs in which risks outweigh benefits should be reserved only for specific diseases or populations. The post-marketing of drugs needs to include doctors and patients to make informed decisions. The drug development process cannot study all the adverse events and ADRs of drugs. This needs separate trials specifically designed to detect ADRs, preferably involving randomization with adverse events of concern as the primary outcome measure. Reporting of ADRs has become common practice worldwide, and future studies must focus on implementing new ideas and perhaps develop AI-based models for easy detection of adverse events.
